# Prevalence of depressive and anxiety disorders in patients with glaucoma: a cross-sectional study

**DOI:** 10.5935/0004-2749.20210006

**Published:** 2025-02-02

**Authors:** Ricardo Yuji Abe, Ludmila Nascimento P. Silva, Daniela Mendes Silva, José Paulo Cabral Vasconcellos, Vital Paulino Costa

**Affiliations:** 1 Hospital Oftalmológico de Brasília, Brasília, DF, Brazil; 2 Hospital das Clínicas, Universidade Estadual de Campinas, Campinas, SP, Brazil

**Keywords:** Glaucoma, Depression/epidemiology, Anxiety/epidemiology, Cross-sectional studies, Glaucoma, Depressão/epidemiologia, Ansiedade/ epidemiologia, Estudos transversais

## Abstract

**Purpose:**

Our goal was to analyze the prevalence of depression and anxiety among patients with glaucoma and to identify risk factors related to these disorders.

**Methods:**

A cross-sectional study was carried out between August 2016 and August 2017 at the Hospital das Clínicas of Universidade Estadual de Campinas and at the Hospital Oftalmológico de Brasília to evaluate the prevalence of depressive and anxiety disorders among patients diagnosed with glaucoma. All patients underwent a complete ophthalmologic examination with standard automated perimetry to confirm the diagnosis of glaucoma. All participants were asked to complete the Hospital Anxiety and Depression Scale questionnaire.

**Results:**

One hundred and twenty-nine patients were included in the study. Seventy-four were men (57.36%) and 55 (42.64%) were women. The mean age of the patients was 70.14 ± 15.8 years. Ninety participants were white (69.77%) and 38 (29.46%) were black. The study demonstrated a prevalence of depression and/or anxiety at 10.08%. Logistic regression revealed that women were at higher risk for anxiety and/or depression (OR: 5.25, p=0.015) and patients with a larger number of co-morbidities also were at higher risk for anxiety and/or depressive disorders (OR: 2.82, p=0.038).

**Conclusion:**

A sig nifi cant proportion of patients with glaucoma present with depression and/or anxiety. Females and patients with co-morbidities are at greater risk for these disorders.

## Abstract (pt)

**Objetivo:**

Avaliar a prevalência de transtornos de depressão e ansiedade em pacientes com glaucoma e identificar fatores de riscos associados.

**Métodos:**

Estudo transversal em pacientes com glaucoma, avaliados durante Agosto de 2016 e Agosto de 2017 no Hospital das Clínicas da Universidade de Campinas e no Hospital Oftalmológico de Brasília. Todos pacientes foram submetidos à exame oftalmológico completo para confirmar o diagnóstico de glaucoma. Todos pacientes preencheram o questionário “Hospital Anxiety and Depression Scale”.

**Resultados:**

Foram incluídos 129 pacientes no estudo, sendo 74 homens (57.36%) e 55 (42.64%) mulheres, 90 pacientes eram brancos (69.77%) e 38 (29.46%) eram negros. A idade média foi de 70.14 ± 15.8 anos. O estudo demonstrou uma prevalência de 10.08% de transtornos depressivo e/ou ansiedade. A regressão logística demonstrou que mulheres apresentam maior risco de desenvolver transtornos depressivos e/ou ansiedade (Risco relativo: 5.25, p=0.015), assim como pacientes com maior número de co-morbidades clínicas (Risco relativo: 2.82, p=0.038).

**Conclusão:**

Uma proporção significativa dos pacientes com glaucoma podem apresentar transtornos de depressão e/ ou ansiedade. Pacientes com glaucoma do sexo feminino e que apresentem maiores co-morbidades clínicas apresentam maior risco de apresentar esses transtornos.

## INTRODUCTION

Glaucoma is the most common cause of irreversible blindness in the world; the diagnosis of glaucoma has been associated with anxiety and depression, the two of the most prominent and pervasive psychiatric disorders^(1,2)^. As in psychiatric disorders, age is an important risk factor for the development of glaucoma^(3)^. Mental health may have an impact on clinical factors such as adherence to antihypertensive medication and treatment follow-up^(4)^. By contrast, the prevalence of depressive disorders in patients with glaucoma may be high due to the fact that visual loss leads to a significant decrease in quality of life^(5,6)^.

Studies have already documented a relationship between psychiatric disorders and glaucoma using depression and anxiety scales; the prevalence of these specific psychiatric disorders is higher among glaucoma patients compared to control groups matched by both sex and age^(7)^. Previous studies have estimated that the prevalence of depressive symptoms in patients with glaucoma is ~10 - 12%. When compared to healthy control subjects, the prevalence of depression was on average 32% higher in patients diagnosed with glaucoma^(1,4)^. Interestingly, Mabuchi et al.^(7)^ found that visual acuity and severity of visual field defects were not associated with severity of the depressive disorder. However, Diniz-Filho et al.^(8)^ reported that progressive visual field loss was associated with the prevalence of depressive symptoms in patients with glaucoma.

Several tools are used to identify patients with depression and anxiety^(9,10)^. The Hospital Anxiety and Depression Scale (HADS) which is divided into anxiety and depression subscales, was developed by Zigmond and Snaith in 1983 to identify anxiety and depression among patients in non-psychiatric hospital clinics^(11)^. HADS is used widely and has been shown to be effective at assessing symptoms of anxiety and depression in primary care clinic patients as well as in the general population^(12,13)^.

The primary objective of this study was to analyze the prevalence of depressive and /or anxiety disorders using the HADS instrument in a group of Brazilian patients diagnosed with glaucoma and to identify potential disease-related risk factors.

## METHODS

### Study design

This cross-sectional study was conducted between August 2016 and August 2017 in accordance with the ethical principles of the Helsinki Declaration and the principles of the current Good Clinical Practices. The study protocol was approved by the Clinical Research Ethics Committee of the Faculty of Medical Sciences of the State University of Campinas. All patients provided written informed consent. The study was carried out by all authors; the second author ensured data integrity and accuracy and study analysis.

### Study population

Patients were selected for study enrollment at the glaucoma outpatient clinic of the Hospital das Clínicas of the State University of Campinas and Hospital Oftalmológico de Brasília. Inclusion criteria included typical glaucomatous changes in the optic nerve (cup/disc ratio >0.6, cup/disc ratio asymmetry >0.2, atrophy of the nerve fiber layer, localized loss of neuro-retinal tissue). Enrollees also displayed characteristic defects identified by standard automated perimetry (SAP), including at least two consecutive abnormal SAP results at baseline with corresponding optic nerve damage in at least one eye. Abnormal SAP results were defined as a pattern standard deviation with p<0.05, glaucoma hemi-field test results outside normal limits, or both findings^(14)^.

All patients underwent a complete ophthalmologic examination, including logMAR best corrected visual acuity, slit lamp biomicroscopy, Goldmann aplannation tonometry, Possner lens gonioscopy, and examination of the fundus through a dilated pupil. Patients with retinal diseases such as age macular degeneration were excluded. All patients also underwent standard automated perimetry (Humphrey, Sita Standard 24-2) and filled out questions in the previously Portuguese-language-validated HADS instrument together with a form requesting socio-demographic data^(15)^.

The stage of glaucomatous damage was defined according to Hodapp, Parish and Anderson criteria^(14)^. We classified patients with advanced glaucoma when the mean deviation (MD) was <-12dB. We also identified patients with low vision according to International Classification of Diseases and Related Health Problems from World Health Organization, including patients with corrected visual acuity that was <20/70^(16)^.

We also reviewed past and present medical histories for any of the following co-morbidities: diabetes mellitus, arthritis, hypertension, heart disease, depression, asthma, and cancer^(5)^. A simple summation score was used to generate a co-morbidity index^(17)^.

Data on gender, age, race, level of education, previous glaucoma surgery, use of antihypertensive eye drops, use of psychoactive substances and past or present psychiatric disorders and/or a specific history of depression and/or anxiety were also documented.

The Hospital Anxiety and Depression Scale (HADS) includes 14 items, seven of which are focused on the assessment of anxiety and seven on depression. Each of the items score from 0 to 3, with a maximum score of 42 points, 21 points for each scale. All patients who had a score greater than or equal to 12 on the depression or the anxiety scale were referred to psychiatric services for follow-up. All forms administered personally by the investigators (RYA, LNS and DMS) to provide support and ensure full comprehension of the questionnaire.

### Statistical analysis

The normality of the sample distribution was evaluated by the inspection of histograms. Student’s t test was used for statistical evaluation continuous variables and for samples that showed a normal distribution. Categorical variables were compared using the Chi-square or Fisher’s test.

We performed a logistic regression to define the odds ratio of depression and/or anxiety disorder. We used as dependent variable patients who had a score greater than or equal to 12 on the HADS scale (coded as 0 or 1). The following parameters were used as independent variables (adjusted for continuous and categorical data): age, gender, race, marital status, education, family income, employment status, co-morbidity index, number of glaucoma eye drops used per day, presence of advanced glaucoma in one or both eyes, the degree of mean eye deviation, visual acuity, presence of low vision in one or both eyes, pseudophakia, use of topical beta-blockers and history of glaucoma surgery. Values of p<0.05 were considered statistically significant. All data analyses were performed using the statistical program Stata (version 13; StataCorp LP, CollegeStation, TX).

## RESULTS

From January 2016 to January 2017, we identified 129 patients diagnosed with glaucoma who were eligible for the study and were included in the analysis. Seventy-four were men (57.4%) and 55 (42.6%) were women. The mean age (± SD) of the patients was 70.14 ± 15.8 years. Ninety participants were white (69.77%) and 38 (29.46%) were black. Table 1 summarizes the clinical and demographic characteristics of the individuals included in the study.

**Table 1:** Demographic characteristics of study subjects

Characteristic	Value
Age, years	
Mean ± SD	70.14 ± 15.8
Gender n (%)	
Male	74 (19.38%)
Female	55 (42.64%)
Race n (%)	
White	90 (69.77%)
Black	38 (29.46%)
Asian	1 (0.78%)
Income n (%)	
Up to R$ 1499,00	64 (50.39%)
R$1.450,00- R$2.989,00	27 (21.26%)
R$2.990,00 - R$7.249,00	20(15.75%)
R$7.250,00-R$14.499,00	7(5.51%)
Above R$14.499,00	9 (7.09%)
Marital status n (%)	
Single	25 (19.38%)
Married	84 (65.12%)
Widower	18(13.95%)
Divorced	2 (1.55%)
Education η (%)	
Illiterate	20(15.50%)
1^st^ degree incomplete	46 (35.66%)
1^st^ degree complete	20(15.50%)
2^nd^ degree incomplete	10(7.75%)
2^nd^ degree complete	16 (12.40%)
Superior degree incomplete	4(3.10%)
Superior degree complete	13 (10.08%)
Employment status n (%)	
Employee	23 (17.83%)
Unemployed	9 (6.98%)
Retired	97 (75.19%)

Best corrected logMAR visual acuities were 0.32 ± 0.52 and 0.93 ± 0.99 in the better and worse eye, respectively. Mean deviations in the better and worse eyes were -5.27 ± 6.34 dB and -11.32 ± 9.24 dB, respectively (Table 2). Among the 129 patients, 13 (10.1%) presented depression and/or anxiety; of this group, 3 (2.3%) presented with depression only, 6 (4.7%) presented with anxiety only and 4 (3.10%) presented with both depression and anxiety (Table 2).

**Table 2:** Clinical characteristics of study subjects

Characteristic	Value
Mean Deviation (mean ± SD) Better eye Worse eye	-5.27 ± 6.34 -11.32 ± 9.24
BCVA (mean ± SD) Better eye Worse eye	0.32 ± 0.52 0.93 ± 0.99
Co-morbidity Index (mean ± SD)	1.19 ± 0.74
Advanced glaucoma in both eyes n (%) Yes	10 (7.75%)
Advanced glaucoma in one eye n (%) Yes	46 (35.66%)
Low Vision vision in both eyes n (%) Yes	9 (6.98%)
Low Vision vision in one eye n (%) Yes	32 (32.56%)
Depression and/or anxiety n (%) Yes	13 (10.08%)
Depression n (%) Yes	3 (2.33%)
Anxiety n (%) Yes	6 (4.65%)
Depression and anxiety n (%) Yes	4(3.10%)

SD= standard deviation; MD= mean deviation; BVCA= best corrected logMAR visual acuity.

Logistic regression revealed that gender and co-morbidity indices were risk factors for anxiety and/or depressive disorders in patients with glaucoma (Table 3). Female patients had an increased risk for anxiety and/ or depression compared to male patients (OR: 5.25, p=0.015) and patients with more co-morbidities also had a higher risk for have anxiety and/or depression (OR: 2.82, p=0.038).

**Table 3:** Predictors of anxiety and/or depressive disorders from logistic regression

Predictors	Odds ratio	p-value
Age	1.00	0.851
Gender	**5.25**	**0.015**
Race	1.12	0.118
Marital status	0.68	0.430
Family Income	0.59	0.128
Level of Instruction	0.94	0.750
Employment status	0.85	0.584
Co-morbidity Index	**2.82**	**0.038**
Glaucoma eyedrops	0.91	0.704
Advanced Glaucoma in both eyes	2.45	0.292
Advanced Glaucoma in one eye	0.78	0.698
Low vision in both eyes	1.12	0.915
Low vision in one eye	0.91	0.885
MD Worse eye	0.99	0.843
BCVA Worse eye	0.98	0.950
Pseudophakia	0.38	0.160
Use of Beta-blocker	1.12	0.852

MD= mean deviation; BCVA= best corrected logMAR visual acuity.

## DISCUSSION

In this study, we determined the prevalence of depression and/or anxiety disorders among 129 patients diagnosed with glaucoma to be 10.1%. We also found that female glaucoma patients and glaucoma patients with a larger number of associated co-morbidities were at greater risk for depression and/or anxiety disorders. To our knowledge, this is the first study to examine the association between glaucoma and anxiety/depression disorders in Brazil, as well as to investigate potential risk factors.

Glaucoma is a chronic degenerative disease and the leading cause of irreversible blindness worldwide^(18)^. Considering the asymptomatic nature of the disease and its potentially devastating outcomes, and likewise the economic impact related to the cost and side effects of antihypertensive medications used to control it, glaucoma might cause or aggravate pre-existing psychological burdens in this patient cohort^(19)^. Anxiety and depression are common psychological disorders that are important public health problems^(20,21)^.

Previous studies have shown that patients with glaucoma are more likely to suffer from both anxiety and depression^(7,22,23)^. Our findings reveal that the prevalence of depression and/or anxiety in this patient cohort was 10.1%; 2.33% reported only depression, 4.65% reported only anxiety and 3.10% presented with both depression and anxiety. These findings are consistent with those reported previously that identified higher rates of depression and anxiety among glaucoma patients. Rezapour et al.^(24)^ reported the prevalence of depression and anxiety among 293 glaucoma patients at 6.6% and 5.3%, respectively. Mabuchi et al.^(1)^ found that, among 230 patients undergoing treatment for glaucoma, 13.0% and 10.9% of also experienced anxiety and depression, respectively.

It is also critical to consider these observations from the opposite perspective. It is also possible that the presence of depressive symptoms in patients with glaucoma may lead to poor compliance with the medication regimen^(25,26)^. As such, it will be critical to provide these patients with adequate psychological care not only to improve quality of life but also to ensure medication compliance^(25)^. In order to detect, prevent and treat anxiety and depression that may coexist among those with glaucoma, it is also important to have some comprehension of the risk factors associated with these psychological disorders. Our findings suggest that female gender and patients with a higher number of co-morbidities are at greater risk for a diagnosis of depression and/or anxiety.

We found that female patients had a 5.25-fold higher chance of succumbing to depression and/or anxiety disorders when compared to age-matched male patients (p=0.015). Among the 55 women from our sample, 10 presented with anxiety and/or depression, while only 3 of 74 men were diagnosed with the same disorders. Lim et al.^(22)^ also reported that female glaucoma patients were more likely to suffer from depression. Tastan et al.^(27)^ also found that the risk of anxiety in women with glaucoma was 7.5 times higher than in men. Society-driven risk factors for anxiety and depression in women are likely have a biological origin; among these traits, we consider differences in physical strength and related personality traits^(28)^.

Our study also demonstrated that patients with more co-morbidities were at higher risk for anxiety and/or depression (OR=2.82, p=0.038). The co-morbidity index was created using a simple summation score from questions regarding present conditions and/or history of conditions such as: diabetes mellitus, arthritis, high blood pressure, heart disease, depression, asthma, and cancer^(5,17)^. Our sample included 25 patients with no co-morbidities; no depression or anxiety were detected among members of this patient cohort. In the group of patients with one co-morbidity (49 patients), 5 patients (10.2%) were diagnosed with anxiety and/or depression. Finally, in the group with 2 or more co-morbidities (42 patients), 8 (19%) presented with anxiety and/or depression.

Previous studies have revealed that depressive disorders play important roles in the etiology, course, and outcomes associated with chronic diseases^(20)^. In fact, patients with chronic medical illnesses are also known to be at high risk for both depression and anxiety^(20,21)^.

Interestingly, objective measures of visual acuity and severity of glaucoma as defined by the mean deviation from standard automated perimetry were not at all predictive of depression or anxiety disorders. This may be explained due to the fact that our sample did not include a large proportion of patients with low vision and advanced glaucoma. However, earlier studies have demonstrated that objective measures of visual function may not be directly associated with the prevalence of psychiatric disorders^(4)^. These results suggest that the asso ciation between glaucoma and depression or anxiety is complex and reflects the perceptions of patients and the subjective experiences of their illness rather than conventional objective measures, such as visual acuity or visual field.

We recognize that our study has several limitations. First, the study design is cross-sectional and there was no follow-up phase. Hence, we were not able to document the psychological changes that may become more or less evident over time. Second, our study did not include a control group which would have permitted us to determine whether the prevalence of psychiatric disorders is actually higher in the glaucoma group. We did not explore the duration of glaucoma in order to evaluate the possibility that length of disease might correlate with a higher prevalence of psychiatric disorders. Finally, although the HADS scale is easy and convenient for testing purposes, the questionnaire is not comparable to a formal psychiatric diagnosis from a mental health specialist.

In summary, this cross-sectional study revealed that a significant proportion of glaucoma patients also present with depression and/or anxiety disorders. We found that females and patients with co-morbidities were at greater risk for depression and or anxiety. Ophthalmologists should make an attempt to recognize these psychiatric disorders in patients with glaucoma and refer them to appropriate care; this will provide dramatic improvements in the patient’s quality of life^(26)^. Ophthalmologists should also provide accurate and appropriate information about glaucoma to their patients in order to prevent the development of excessive and inappropriate anxiety and depression^(29)^. Furthermore, longitudinal studies should be conducted with a larger sample size and with reliable tools that were designed to assess psychological disorders. This might be accompanied by an investigation into whether the treatment of these psychiatric disorders may result in an improved the quality of life for patients with glaucoma and promote increased adherence to anti-glaucomatous treatment.
